# Supplementary material for: The effect of school smoke-free policies on smoking stigmatization: A European comparison study among adolescents
Source: PLoS One. 2020 Jul 14;15(7):e0235772. doi: 10.1371/journal.pone.0235772 (PMC7360046; doi:10.1371/journal.pone.0235772)
Supplement: S1 Table — (DOCX) [file pone.0235772.s001.docx]

**S1 Table Participation rates among students by city, SILNE-R study in seven EU cities, 2016**

| Cities – Countries | **2016** | | | | |
| --- | --- | --- | --- | --- | --- |
|  | **N** | **Participation rate** (%) | **Std**  (%) | **Min**  (%) | **Max**  (%) |
| **Students sample** |  |  |  |  |  |
| Namur – Belgium (N_schools_ = 7) | 1939 | 84.1 | 0.76 | 82.5 | 85.5 |
| Tampere – Finland (N_schools_ = 9) | 1733 | 87.1 | 0.75 | 85.5 | 88.5 |
| Hanover – Germany (N_schools_ = 12) | 1497 | 65.8 | 0.99 | 63.8 | 67.7 |
| Latina – Italy (N_schools_ = 7) | 1982 | 78.9 | 0.81 | 77.3 | 80.5 |
| Amersfoort – The Netherlands (N_schools_ = 6) | 1858 | 84.9 | 0.76 | 83.4 | 86.4 |
| Coimbra – Portugal (N_schools_ = 6) | 1862 | 76.2 | 0.86 | 74.5 | 77.8 |
| Ireland – Dublin (N_schools_ = 8) | 2120 | 80.3 | 0.77 | 78.7 | 81.7 |
