# Supplementary material for: The effect of school smoke-free policies on smoking stigmatization: A European comparison study among adolescents
Source: PLoS One. 2020 Jul 14;15(7):e0235772. doi: 10.1371/journal.pone.0235772 (PMC7360046; doi:10.1371/journal.pone.0235772)
Supplement: S2 Table — (DOCX) [file pone.0235772.s002.docx]

**S2 Table: STP dimensions construction, SILNE-R study in seven EU cities**

| Dimensions | Source | Questions | Score |
| --- | --- | --- | --- |
| Comprehensiveness | Staff | 1. Where and to whom the policy applies   School buildings, for students  School grounds, for students  School bus, for students  School-sponsored events, for students  School buildings, for staff  School grounds, for staff  School bus, for staff  School-sponsored events, for staff  School buildings, for visitors  School grounds, for visitors  School bus, for visitors  School-sponsored events, for visitors   1. When the policy applies   During school hours, for students  During non-school hours, for students  During school hours, for staff  During non-school hours, for staff  During school hours, for visitors  During non-school hours, for visitors   1. Smoking rooms   No smoking room for students  No smoking room for staff  No smoking room for visitors | 1  1  1  1  1  1  1  1  1  1  1  1  1  1  1  1  1  1  1  1  1 |
|  | Student | In 2013: Is there a rule against tobacco in your school?  There is no rule; I don't know  There is a rule but it isn't enforced; there is a rule and it is sometimes enforced; there is a rule and it is strictly enforced  -----  In 2016: Are students allowed to smoke on the school premises?  Yes, students are allowed to smoke in certain areas; yes, students are allowed to smoke anywhere on the school premises; I don't know  No, students are not allowed to smoke. This rule is strictly enforced; no, students are not allowed to smoke. But this rule is not strictly enforced | 0  1  0  1 |
| Enforcement | Staff | What were the consequences for students who were caught violating your school’s smoking rules since September?  No rule violation  or  Encouraged, but not required, to participate in an assistance, education, or quit-smoking programme; required to participate in an assistance, education, or quit-smoking programme  Referred to a school administrator; referred to a school counsellor; warning issued; tobacco confiscated; parents informed; assigned additional classwork; assigned to help around the school; fine; detention; suspension; expulsion | 1  or  2/3  1/3 |
|  | Student | In 2013: Is there a rule against tobacco in your school?  There is no rule; there is a rule but it isn't enforced; there is a rule and it is sometimes enforced; I don't know  There is a rule and it is strictly enforced  -----  In 2016: Are students allowed to smoke on the school premises?  Yes, students are allowed to smoke in certain areas; yes, students are allowed to smoke anywhere on the school premises; no, students are not allowed to smoke. But this rule is not strictly enforced; I don't know  No, students are not allowed to smoke. This rule is strictly enforced | 0  1  0  1 |
| Communication | Staff | 1. Means of communication of the rule to students   In writing in a student diary  In writing in a school handbook  In writing in a school newsletter  Verbally, in discussions with students  Posted on school website(s)  On display at school  E-mail   1. procedures for informing…   Students about the rules related to cigarette-smoking  Students about the consequences of violating the rules  Parents about the rules related to cigarette-smoking  Parents about the consequences of violating the rules  Staff about the rules related to cigarette-smoking  Staff about the consequences of violating the rules  Visitors about the rules related to cigarette smoking  Visitors about the consequences of violating the rules | 1  1  1  1  1  1  1  1  1  1  1  1  1  1  1 |
