# Supplementary material for: The effect of school smoke-free policies on smoking stigmatization: A European comparison study among adolescents
Source: PLoS One. 2020 Jul 14;15(7):e0235772. doi: 10.1371/journal.pone.0235772 (PMC7360046; doi:10.1371/journal.pone.0235772)
Supplement: S1 File — (DOCX) [file pone.0235772.s003.docx]

**S1 File: Ethical approvals**

In each country, ethical approval was requested and obtained from the local or national ethical committees listed below. Following the recommendations of the European Commission, national and local organizations, the datasets generated in this study are not publicly available. Making all the data available would contravene the Consortium Agreement that was developed and signed before the start of the SILNE-R project, specifically with regards to the ownership and use of the project output. Furthermore, we would like to retain control over the use of the data in order to avoid it being used inappropriately by the tobacco industry. In 2021, however, any person can apply for access to the data through the SILNE R consortium (the data owner). The contact person, as data holder, is Anton E Kunst, a.e.kunst@amsterdamumc.nl. Within the Institute, the two contact persons are: Regina Below, from the research department, regina.below@uclouvain.be; and Alaa Mahboub, for technical support, alaa.mahboub@uclouvain.be.

Belgium

2013: Commission d’Ethique Biomédicale, reference number: 2012/09OCT/461.

2016: Comité d’éthique Hospitalo-Facultaire des Cliniques Universitaires Saint-Luc, reference number : 2012/09OCT/461. N° enregistrement belge B403201215182.

Germany

2013: Ethics committee, Medical Faculty, Martin-Luther-University Halle-Wittenberg, Germany, reference number: 2012-112, approved on 13/12/2012.

2016: Ethical approval MLU Halle-Wittenberg: 2016-90 hm-bü. Supervisory school authority Han(n)over: H 1 R b - 81402 - 55 - 2016. Supervisory school authority Lüneburg (Celle): LG 1 R.22 – 503000.

Portugal

2013: General Directorate for Education (Direção Geral da Educação), reference number: 0338600001, approved on 02/11/2012.

2016: General Directorate for education, reference number 0338600002, approved on 26/06/2016.

The Netherlands

2013: Medical Ethical Committee of the AMC, reference number: W12_256#12.17.0290.

2016: The Medical Ethics Review Committee of the Academic Medical Centre confirmed that the Medical Research Involving Human Subjects Act (WMO) does not apply to the SILNE-R study and that official approval was therefore not required: reference number W16_252 # 16.297, 11 August 2016

Finland

2013: Ethics Committee of the Tampere region. Favourable Statement reference number: 10/2012.

2016: Ethics Committee of the Tampere Region, Statement 29/2016.

Italy

2013: Ethics committee, Azienda Unità Sanitaria, Locale Frosinone, Italy, reference number: 862, approved on 13/11/2012.

2016: Ethical Committee ‘Lazio 2’, protocol number 0068451/2016.
