# Supplementary material for: The effect of school smoke-free policies on smoking stigmatization: A European comparison study among adolescents
Source: PLoS One. 2020 Jul 14;15(7):e0235772. doi: 10.1371/journal.pone.0235772 (PMC7360046; doi:10.1371/journal.pone.0235772)
Supplement: S2 File — (PDF) [file pone.0235772.s004.pdf]

SURVEY

SILNE-R

You, your school, your health

TOBACCOFREE RESEARCH INSTITUTTE IRELAND

BEFORE YOU START, PLEASE READ THIS.

This survey is part of an international study called SILNE-R. Most of the questions are about smoking, but there are also questions about your friends, family, school life, and health. This questionnaire will be completed by more than 10,000 students like you, in seven European countries.

This is an anonymous survey. **All the information will be kept confidential and no names will be used**. No one will know your answers and there will be no consequences for your school work.

Taking part is completely voluntary. You are free not to participate or to skip any question you don't want to answer.

Please mark your answer for each question by ticking the box. There is no right or wrong answer. It is important that you answer as truthfully and openly as possible.

Please put your completed questionnaire into the enclosed envelope and seal it yourself. One of the survey team will collect the envelopes after you have filled in the questionnaire.

If you have a question, please raise your hand and one of the survey team will assist you.

I have read the above information and I agree to take part in the SILNE-R study. ◯ YES

Please write in the squares below your unique identifier number which has been provided to you on a separate sheet/ or which is being projected onto the screen by a member of staff. Do not write your name on this page, please use the assigned identifying number.

☐☐☐☐

THANK YOU SO MUCH FOR TAKING PART!

1. In the box below, write the code that appears opposite your name.

1. ☐☐☐☐

PEER GROUP

The next few questions are about your friends.

2. Using the student list provided, which of your schoolmates do you prefer to work with or ask for advice, for example on homework or on an assignment. Do not write their names on this questionnaire, but look up their name on the list provided, and write their code into the adjacent box.

You can write up to five codes into the boxes

Code

1. ☐☐☐☐
2. ☐☐☐☐
3. ☐☐☐☐
4. ☐☐☐☐
5. ☐☐☐☐

3. Using the student list provided, who are your best friends? You can include friends (of any gender) and a girlfriend/ boyfriend if you have one. Do not write their names on this questionnaire but look up their name on the list provided, and write their code into the adjacent box.

You can write up to five codes into the boxes.

Code

1. ☐☐☐☐
2. ☐☐☐☐
3. ☐☐☐☐
4. ☐☐☐☐
5. ☐☐☐☐

4. Would you say that your closest friends are…?

Tick one box.

1. ◯ Mostly in my school
2. ◯ I have as many friends inside as outside my school
3. ◯ Most of my friends are outside my school
4. ◯ I have no close friends

DEMOGRAPHICS

The next few questions ask for some background information about you.

5. How old are you today?

Tick one box.

1. ◯ 12 years
2. ◯ 13 years
3. ◯ 14 years
4. ◯ 15 years
5. ◯ 16 years
6. ◯ 17 years
7. ◯ 18 years
8. ◯ 19 years or older

6. Are you …

Tick one box.

1. ◯ Female
2. ◯ Male

7. In which country were you born?

Tick one box.

1. ◯ In this country ‣ GO TO QUESTION **9.**
2. ◯ Another country, name of country: ____________________ ‣ GO TO QUESTION **8.**

8. If you were born in another country, in what year did you move to this country?

Write the year in the box below.

1. Year ☐

GENERAL HEALTH

The next few questions are about your health and your body.

9. Would you say your health is…? *Tick one box.*

1. ◯ Excellent
2. ◯ Good
3. ◯ Fair
4. ◯ Poor

10. Thinking about last week…

For each question, please give the answer that comes closest to the way you have been feeling. Tick one box on each line.

	Never (I)	Seldom (II)	Quite often (III)	Very often (IV)	Always (V)
1. Have you felt full of energy?	◯	◯	◯	◯	◯
2. Have you felt sad?	◯	◯	◯	◯	◯
3. Have you felt lonely?	◯	◯	◯	◯	◯
4. Have you had enough time for yourself?	◯	◯	◯	◯	◯
5. Have you been able to do the things that you want to do in your free time?	◯	◯	◯	◯	◯
6. Have your parent(s) treated you fairly?	◯	◯	◯	◯	◯
7. Have you had fun with your friends?	◯	◯	◯	◯	◯
8. Have you been able to pay attention?	◯	◯	◯	◯	◯

	Not at all (I)	Slightly (II)	Moderately (III)	Very much so (IV)	Extremely (V)
9. Have you felt fit and well?	◯	◯	◯	◯	◯
10. Have you got on well at school?	◯	◯	◯	◯	◯

SILNE-R

11. The next question is about your physical activity.

This includes physical activity during physical education class, lunch, evening, and spare time. Physical activities are jogging, cycling, team sports, fast dancing and any other physical activities that increase your heart rate and make you breathe hard or sweat.

Tick one cell per day.

	None (I)	30 minutes (II)	1 hour (III)	1 hour and half (1.5 hours) (IV)	2 hours (V)	2 hours and half (2.5 hours) (VI)	3 or more hours (VII)
1. Monday	◯	◯	◯	◯	◯	◯	◯
2. Tuesday	◯	◯	◯	◯	◯	◯	◯
3. Wednesday	◯	◯	◯	◯	◯	◯	◯
4. Thursday	◯	◯	◯	◯	◯	◯	◯
5. Friday	◯	◯	◯	◯	◯	◯	◯
6. Saturday	◯	◯	◯	◯	◯	◯	◯
7. Sunday	◯	◯	◯	◯	◯	◯	◯

12. Thinking back over the last 12 months, how often did you have a drink of alcohol (more than just a sip)?

"A drink of alcohol" means: 1 regular-sized bottle, can, or glass of beer; 1 glass of wine; 1 shot of spirits (rum, whisky, etc.); or a mixed drink (1 shot of spirits with juice, energy drink, etc.).

Tick one box.

1. ◯ I did not have a drink of alcohol in the last 12 months
2. ◯ Less than once a month
3. ◯ Once a month
4. ◯ 2 or 3 times a month
5. ◯ Once a week
6. ◯ Most days of the week
7. ◯ Daily

13. In the last 12 months, how often did you have 5 or more alcoholic drinks on one occasion?

Tick one box.

1. ◯ I did not have 5 or more drinks on one occasion in the last 12 months
2. ◯ Less than once a month
3. ◯ Once a month
4. ◯ 2 or 3 times a month
5. ◯ Once a week
6. ◯ Twice or more a week

14. In the last 12 months, how often did you go out to bars/clubs?

Tick one box.

1. ◯ Never
2. ◯ Once a month or less
3. ◯ 2-3 times a month
4. ◯ Once a week
5. ◯ More than once a week

15. Thinking back over the last 12 months, how often did you use marijuana or cannabis? (a joint, pot, weed, hash...).

Tick one box.

1. ◯ I have never used marijuana
2. ◯ I have used marijuana but not in the last 12 months
3. ◯ Less than once a month
4. ◯ Once a month
5. ◯ 2 or 3 times a month
6. ◯ Once a week
7. ◯ Most days of the week
8. ◯ Daily

SMOKING

The next few questions are about smoking (including cigarettes, roll your own tobacco (rollies), cigars, and cigarillos or little cigars).

These questions do not concern e-cigarettes – there will be questions about them later.

There is a lot of talk these days about the use of tobacco, but very little accurate information. We still have a lot to learn about the actual experiences and attitudes of people your age.

16. Have you ever tried cigarette smoking, even just a few puffs?

Tick one box.

1. ◯ No ‣ GO TO QUESTION **28.**
2. ◯ Yes

EVERTRIERS and SMOKERS

17. How old were you when you first tried smoking cigarettes, even just a few puffs?

Tick one box.

1. ◯ I have never smoked
2. ◯ 9 years or younger
3. ◯ 10 years
4. ◯ 11 years
5. ◯ 12 years
6. ◯ 13 years
7. ◯ 14 years
8. ◯ 15 years
9. ◯ 16 years
10. ◯ 17 years
11. ◯ 18 years or older

18. How many cigarettes have you smoked during the last 30 days?

Tick one box.

1. ◯ I have never smoked
2. ◯ None
3. ◯ 1 to 2 cigarettes during the last 30 days
4. ◯ 1 to 2 cigarettes per week
5. ◯ 3-7 cigarettes per week
6. ◯ 1 to 5 cigarettes per day
7. ◯ 6 to 10 cigarettes per day (about 1/2 a packet total)
8. ◯ 11 to 20 cigarettes per day (about 1 packet total)
9. ◯ 21 to 30 cigarettes per day
10. ◯ More than 30 cigarettes per day

19. Thinking back 12 months ago, has your tobacco consumption changed?

Tick one box .

1. ◯ Yes, I started to smoke
2. ◯ Yes, I smoke more than last year
3. ◯ Yes, I smoke less than last year
4. ◯ No, I smoke as much as last year
5. ◯ No, I have never smoked

20. Do your parents know that you (have) smoke(d)?

Tick one box .

1. ◯ Yes
2. ◯ No
3. ◯ I don't know
4. ◯ No, I have never smoked

21. Have you ever tried to quit smoking cigarettes? *Tick one box.*

1. ◯ I have never smoked
2. ◯ I have only smoked a few times
3. ◯ I have never tried to quit
4. ◯ I have tried to quit at least once
5. ◯ I have quit smoking ▸ GO TO QUESTION **28.**

22. In the past 30 days, on how many days did you smoke a cigarette (even a puff)?

Tick one box.

1. ◯ None ▸ GO TO QUESTION **28.**
2. ◯ 1 or 2 days
3. ◯ 3-5 days
4. ◯ 6-10 days
5. ◯ 11-20 days
6. ◯ (Almost) every day

23. Where do you usually smoke cigarettes?

Tick all that apply.

1. ◯ At home
2. ◯ On school premises
3. ◯ Just outside school
4. ◯ At a workplace
5. ◯ At friends' houses
6. ◯ In a cafe, pub, nightclub
7. ◯ At a shopping centre
8. ◯ In other public spaces (e.g. parks, street corners)

24. How often do you smoke in school or on its premises?

Tick one box.

1. ◯ Daily
2. ◯ Most days of the week
3. ◯ Some days of the week
4. ◯ Never

25. During the past 30 days (one month), how did you usually get your own cigarettes?

Tick all that apply.

1. ◯ I did not smoke cigarettes during the past 30 days (one month)
2. ◯ I bought them in a shop
3. ◯ I bought them from a vending machine
4. ◯ I bought them from a service station
5. ◯ My parents or siblings gave them to me
6. ◯ Friends gave them to me
7. ◯ I bought them from friends
8. ◯ I bought them from parents or siblings
9. ◯ I bought them on the internet
10. ◯ I asked someone under the age of 18 to buy them for me
11. ◯ I asked an adult I know to buy them for me
12. ◯ I asked an adult I didn't know to buy them for me
13. ◯ I got them some other way

26. In the last 30 days have you bought or tried to buy cigarettes from a shop, vending machine, service station, or on the internet?

Tick one box.

1. ◯ Yes- I bought cigarettes
2. ◯ Yes- I tried to buy cigarettes but was refused
3. ◯ No- I did not try to buy cigarettes
4. ◯ No- I have never tried to buy cigarettes

27. On the whole, do you find it easy or difficult to buy cigarettes from a shop, vending machine, service station, or on the internet?

Tick one box.

1. ◯ Very difficult
2. ◯ Fairly difficult
3. ◯ Fairly easy
4. ◯ Very easy
5. ◯ I don’t buy cigarettes

ALL STUDENTS SHOULD ANSWER THE FOLLOWING QUESTIONS

28. Do you think that you will smoke a cigarette soon?

Tick one box.

1. ◯ Definitely not
2. ◯ Probably not
3. ◯ Probably yes
4. ◯ Definitely yes

29. How difficult do you think it would be for you to get cigarettes if you wanted?

Tick one box.

1. ◯ Very difficult
2. ◯ Fairly difficult
3. ◯ Fairly easy
4. ◯ Very easy

30. If one of your friends offered you a cigarette would you smoke it?

Tick one box.

1. ◯ Definitely not
2. ◯ Probably not
3. ◯ Probably yes
4. ◯ Definitely yes

31. Do any of your best and closest friends smoke cigarettes?

Tick one box.

1. ◯ None of them
2. ◯ Some of them
3. ◯ Most of them
4. ◯ All of them

32. How often do you see students or teachers smoking on school premises?

Tick one box on each line.

	Never (I)	Sometimes	Often (III)	Always (IV)
1. Students	◯	◯	◯	◯
2. Teachers	◯	◯	◯	◯

33. How often do you see people smoking just outside your school?

Tick one box.

1. ◯ Never
2. ◯ Sometimes
3. ◯ Often
4. ◯ Always

34. In the last 6 months, have you seen people smoking in any of the following places?

Tick one box on each line.

	Yes (I)	No (II)	I never go to these places (III)
1. At home	◯	◯	◯
2. At friend's homes	◯	◯	◯
3. Restaurants incl. fast food / diners	◯	◯	◯
4. Bars / cafes / clubs / discos	◯	◯	◯
5. In train / bus station	◯	◯	◯
6. In leisure / sport facilities	◯	◯	◯

SILNE-R

35. In your town, how many people of your age smoke?

Tick one box.

0%	5%	10%	20%	30%	40%	50%	60%	70%	80%	90%	100%
◯	◯	◯	◯	◯	◯	◯	◯	◯	◯	◯	◯
1.	2.	3.	4.	5.	6.	7.	8.	9.	10.	11.	12.

36. In your school, how many people of your age in your school smoke cigarettes?

Tick one box.

0%	5%	10%	20%	30%	40%	50%	60%	70%	80%	90%	100%
◯	◯	◯	◯	◯	◯	◯	◯	◯	◯	◯	◯
1.	2.	3.	4.	5.	6.	7.	8.	9.	10.	11.	12.

37. How would / do these people react if they thought/knew you were smoking?

Tick one box on each line.

	They (would) approve (I)	They (would) not mind (II)	They (would) disapprove a little (III)	They (would) disapprove a lot (IV)
1. Your best friends	◯	◯	◯	◯
2. Your parents	◯	◯	◯	◯
3. Your teachers	◯	◯	◯	◯

38. Smoking increases your chances of…

Tick one box on each line.

	Strongly agree (I)	Agree (II)	Disagree (III)	Strongly disagree (IV)
1. Looking cool	◯	◯	◯	◯
2. Getting into trouble	◯	◯	◯	◯
3. Feeling relaxed	◯	◯	◯	◯
4. Becoming popular	◯	◯	◯	◯
5. Looking grown-up	◯	◯	◯	◯
6. Getting lung cancer	◯	◯	◯	◯
7. Looking masculine	◯	◯	◯	◯
8. Looking feminine	◯	◯	◯	◯
9. Losing weight or keeping thin	◯	◯	◯	◯
10. Having chronic breathing trouble	◯	◯	◯	◯
11. Getting facial wrinkles	◯	◯	◯	◯
12. Appearing sexy/attractive	◯	◯	◯	◯
13. Getting a boy/girlfriend	◯	◯	◯	◯
14. Having less ability to exercise	◯	◯	◯	◯

SILNE-R

39. These questions are about what people think about smokers and smoking.
For each question, please give the answer that comes closest to the way people think.

Tick one box on each line.

	Strongly agree (I)	Agree (II)	Disagree (III)	Strongly disagree (IV)
1. Most people would not hire a smoker to babysit their children	◯	◯	◯	◯
2. Most non-smokers would be reluctant to date someone who smokes	◯	◯	◯	◯
3. Most people believe that smoking is for losers	◯	◯	◯	◯
4. Most people think less of person who smokes	◯	◯	◯	◯

40. Over the last 7 days, on how many days were you in a car with someone who was smoking a cigarette(/s)?

Tick one box.

1. ◯ 0 days
2. ◯ 1 or 2 days
3. ◯ 3 or 4 days
4. ◯ 5 or 6 days
5. ◯ All 7 days
6. ◯ I was not in a car within the last 7 days
7. ◯ I don't know

THE FOLLOWING QUESTIONS CONCERN E-CIGARETTES

41. Which one of the following statements concerning electronic cigarettes best describes you?

Tick one box.

1. ◯ I have never tried an e-cigarette ▸ GO TO QUESTION 44.
2. ◯ I have tried an e-cigarette once or twice
3. ◯ I have tried an e-cigarette more than twice
4. ◯ I use an e-cigarette at least once a month
5. ◯ I use an e-cigarette at least once a week
6. ◯ I use an e-cigarette every day
7. ◯ I don't know what e-cigarettes are ▸ GO TO QUESTION 44.

42. If you have tried/used e-cigarettes, what substance did they contain?

You can tick more than one option.

1. ◯ Liquid with nicotine
2. ◯ Liquid without nicotine
3. ◯ I don't know
4. ◯ I have never tried e-cigarettes

43. When you first tried an e-cigarette, what was your relationship with tobacco?

Tick one box.

1. ◯ I had never smoked tobacco
2. ◯ I had tried tobacco but didn't use it regularly
3. ◯ I smoked tobacco occasionally
4. ◯ I smoked tobacco regularly
5. ◯ I have never tried an e-cigarette

SILNER

YOU AND YOUR SCHOOL

TOBACCO CONTROL AT SCHOOL

The next few questions are about smoking in your school.

44. How do you think the teachers at your school feel about teenagers smoking?

Tick one box.

1. ◯ Most of them approve
2. ◯ Most of them do not mind
3. ◯ Most of them disapprove a little
4. ◯ Most of them disapprove a lot
5. ◯ Don't know

45. Are students allowed to smoke on the school premises?

Tick one box.

1. ◯ No, students are not allowed to smoke. This rule is strictly enforced
2. ◯ No, students are not allowed to smoke. But this rule is not strictly enforced
3. ◯ Yes, students are allowed to smoke in certain areas
4. ◯ Yes, students are allowed to smoke anywhere on the school premises
5. ◯ Don't know

46. Are teachers/staff allowed to smoke on the school premises?

Tick one box.

1. ◯ No, teachers are not allowed to smoke.
2. ◯ Yes, teachers are allowed to smoke in certain areas
3. ◯ Yes, teachers are allowed to smoke anywhere on the school premises
4. ◯ Don't know

47. What would you expect the consequences to have been for students who were caught violating smoking rules in your school during the last six months?

Tick all that apply.

1. ◯ Warning issued (written or verbal)
2. ◯ Educational conversation
3. ◯ Parents informed
4. ◯ Referred to a school administrator or counsellor
5. ◯ Assigned to help around the school
6. ◯ Detention
7. ◯ Suspension
8. ◯ Nothing
9. ◯ Don't know
10. ◯ Other: ___________________________

SCHOOL EXPERIENCE

The next few questions are about your school and some more information about you.

48. When did you start to study in this school?

Tick one box.

1. ◯ School year 2016/2017
2. ◯ School year 2015/2016
3. ◯ School year 2014/2015
4. ◯ School year 2013/2014
5. ◯ Earlier

49. Which of the following best describes your marks during the past year?

Tick one box.

1. ◯ Mostly As (85% or higher)
2. ◯ Mostly As and Bs (84%-75%)
3. ◯ Mostly Bs and Cs (74%- 65%)
4. ◯ Mostly Cs (64%- 55%)
5. ◯ Mostly lower than Cs (54% or lower)

SILNE-R

50. How strongly do you agree or disagree with each of the following?

Tick one box on each line.

	Strongly agree (I)	Agree (II)	Disagree (III)	Strongly disagree (IV)
1. I feel close to people at my school	◯	◯	◯	◯
2. I feel I am part of my school	◯	◯	◯	◯
3. I am happy to be at my school	◯	◯	◯	◯
4. I feel the teachers at my school treat me fairly	◯	◯	◯	◯
5. I feel safe in my school	◯	◯	◯	◯

YOUR FAMILY

The next few questions are about your family and home.

51. In which country was your mother born?

Tick one box.

1. ◯ This country
2. ◯ Another country: Name of country ______________________

52. In which country was your father born?

Tick one box.

1. ◯ This country
2. ◯ Another country: Name of country ______________________

53. What was the highest level of schooling your mother attended?

Tick one box.

1. ◯ Completed primary school or less
2. ◯ Secondary school, not completed
3. ◯ Completed secondary school
4. ◯ College or university, not completed
5. ◯ Completed college or university
6. ◯ I don't know

54. What was the highest level of schooling your father attended?

Tick one box.

1. ◯ Completed primary school or less
2. ◯ Secondary school, not completed
3. ◯ Completed secondary school
4. ◯ College or university, not completed
5. ◯ Completed college or university
6. ◯ I don't know

All families are different (for example, not everyone lives with both their parents, sometimes people live with just one parent, or they have two homes or live with two families) and we would like to know about yours. Please answer the following questions for the home where you live all or most of the time.

55. Which of the following people live in your home (where you live all or most of the time)?

Tick all that apply.

1. ◯ Father
2. ◯ Stepfather
3. ◯ Mother
4. ◯ Stepmother
5. ◯ Brother(s) / Stepbrother(s)
6. ◯ Sister(s) / Stepsister(s)
7. ◯ Grandparent(s)
8. ◯ Other relative(s)
9. ◯ Non-relative(s)
10. ◯ I live alone
11. ◯ I live in a boarding school

56. What language do you speak most often with your parents/guardian?

Tick one box.

1. ◯ English
2. ◯ Irish
3. ◯ Another language (please name the language): ______________________

SMOKING RULES IN YOUR HOME

57. Does any member of your family smoke cigarettes?

Tick one box on each line that applies to your family.

	Yes (I)	No (II)	Stopped smoking (III)	Does not apply (IV)
1. Father	○	○	○	○
2. Stepfather	○	○	○	○
3. Mother	○	○	○	○
4. Stepmother	○	○	○	○
5. Brother(s) / Stepbrother(s)	○	○	○	○
6. Sister(s) / Stepsister(s)	○	○	○	○
7. Grandmother(s)	○	○	○	○
8. Grandfather(s)	○	○	○	○
9. Other relative(s)	○	○	○	○
10. Non-relative(s)	○	○	○	○

58. Is smoking permitted in your home (where you live in all or most of the time)?

Tick one box.

1. ○ No one is permitted to smoke in my home
2. ○ Smoking is permitted only in certain areas of my home
3. ○ Smoking is permitted freely in my home
4. ○ I don't know

SOCIOECONOMIC

59. On average, how much money do you usually get EACH WEEK to spend on yourself?

This money could include pocket money or an allowance; or money from jobs like delivering papers, babysitting etc?

Enter the amount on the following line :

.... **euro**

60. Imagine that this ladder shows how your country society is made up.

At the top of the ladder are the people who are the best off – they have the most money, the highest level of education, and the jobs that bring the most respect. At the bottom of the ladder are the people who are the worst off –they have little or no education, no jobs, or jobs that don't pay much.

Now think about your family. **Please tell us where you think your family would be on this ladder.**

Fill in the circle that best represents where your family would be on this ladder.

10. ○
9. ○
8. ○
7. ○
6. ○
5. ○
4. ○
3. ○
2. ○
1. ○
0. ○

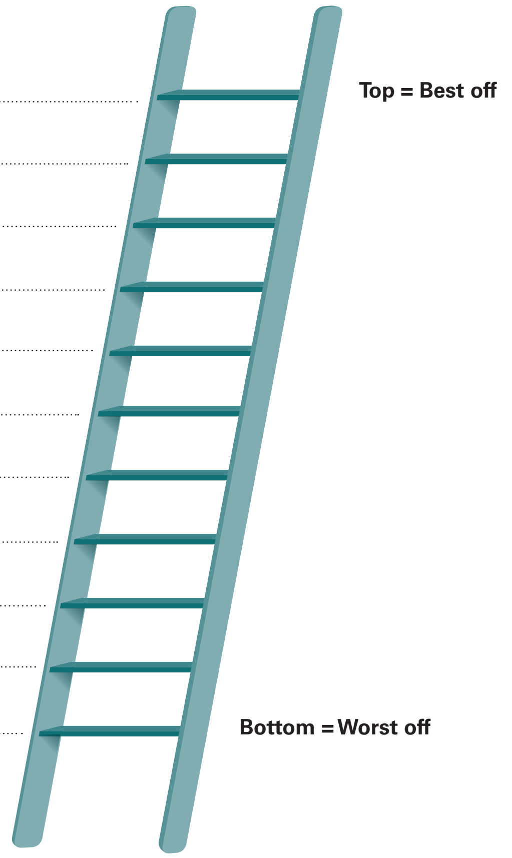

Thank you so much for taking part!

TOBACCOFREE RESEARCH INSTITUTTE
IRELAND

SILNER
